# Supplementary material for: The role of Xist‐mediated Polycomb recruitment in the initiation of X‐chromosome inactivation
Source: EMBO Rep. 2019 Aug 27;20(10):e48019. doi: 10.15252/embr.201948019 (PMC6776897; doi:10.15252/embr.201948019)
Supplement: Supplementary file 1 — Appendix [file EMBR-20-e48019-s001.pdf]

Appendix PDF

Table of Contents:

The Appendix PDF contains Appendix Tables S1, S2 and S3:

- Appendix Table S1 pg 2
- Appendix Table S2 pg 3
- Appendix Table S3 pg 4

Xist mutants	5'end /3'end sgRNAs	Primers to confirm deletion (F/R)	Primers to confirm loss of WT sequence (F/R)
Xist ΔF+B+C	TCACGCAGAAGCCATAATGG/ CTTGAGAGATGATACCTCCA	CTGCTGATCGTTTGGTGCTG/ CAGACCTGTGTTTGCCCCTT	CTGCTGATCGTTTGGTGCTG/ ATCAAGGCGAATCCCGCAAC
Xist ΔF+B	TCACGCAGAAGCCATAATGG/ AGGGCTGGACTGGATTGGGT	TGGTGCTGTGTGAGTGAACC/ TTAGCACTGAATCAATGAAGA	CTGCTGATCGTTTGGTGCTG/ ATCAAGGCGAATCCCGCAAC
Xist ΔB+C	TATAACAGTAAGTCTGATAG/ GTGTATCTTGATTAACATGA	ATGACTGGATGTCAGGAGTA/ CAGACCTGTGTTTGCCCCTT	ATGACTGGATGTCAGGAGTA/ CTGAGTCTTGAGGAGAATCT
Xist ΔB+1/2C	TATAACAGTAAGTCTGATAG/ CATACTGACTTCTAGAGTCA*	ATGACTGGATGTCAGGAGTA/ CAGACCTGTGTTTGCCCCTT	ATGACTGGATGTCAGGAGTA/ CTGAGTCTTGAGGAGAATCT
Xist ΔB	TATAACAGTAAGTCTGATAG/ CTCTAAGTAGAAGTGGGCTT	ATGACTGGATGTCAGGAGTA/ TTAGCACTGAATCAATGAAGA	ATGACTGGATGTCAGGAGTA/ CTGAGTCTTGAGGAGAATCT
Xist ΔC	CTCTAAGTAGAAGTGGGCTT/ GTGTATCTTGATTAACATGA	CCAGGCCCAGATACTTTCAG/ CAGACCTGTGTTTGCCCCTT	TCCATGGACAAGTAAACAAAGA A/ TGTTTGCCCCTTTGCTAAAT

Appendix Table 1 – List of gRNA sequences and primers used for *CRISPR/Cas9* editing of the different *Xist*-TetOP mutants

Primer sequences to confirm deletion and loss of wild-type (WT) allele are displayed for each *Xist* mutant; * highlights for the fact that the 3’end gRNA used to generate ΔB+1/2C was designed to a unique region within the repeat C, but several sequences with only 1 or 2 mismatched can be found within this repeat and are likely to be targeted to generate different types of ΔB+1/2C mutants.

**RT-PCR analysis**	Sequences (F/R)
Xist exon 1- exon 3	GCTGGTTCGTCTATCTTGTGGG / CAGAGTAGCGAGGACTTGAAGAG
Xist before repeat B	ATGACTGGATGTCAGGAGTA / CTGAGTCTTGAGGAGAATCT
Xist before repeat C	TCCATGGACAAGTAAACAAAGAA / TGTTTGCCCCTTTGCTAAAT
Gapdh	AACTTTGGCATTGTGGAAGG **/** ACACATTGGGGGTAGGAACA
18S	GGGAGCCTGAGAAACGGC / GGGTCGGGAGTGGGTAATTT

Appendix Table S2- Primer sequences for RT-PCR analysis of *Xist* mutants (used in Fig. EV1B)

Exon 1 - 5' end Set	Oligo-probes
1	gtctgataacagacctgtgt
2	ttaatggccaatgccttgaa
3	gcagaggtttttggctgaaa
4	agggtagtattaggaccttg
5	aggggacttgaacaactgca
6	gtatgagggtatgggatctt
7	agagaagtggtctcattggt
8	gtgcacacaacaggcacaaa
9	tccaatgcttaggaagaggg
10	tcttcaccagatgcagatta
11	gcacaactgtggacatgaga
12	gtacaggagtcctgatctaa
13	gctttatattagtgagcctc
14	tcttaccttgaaggaccatt
15	agtttgaggaaggggtttca
16	gcctactacaatcagtcatt
17	gtttccttctgtagtgaaca
18	aaggtacatgtttatggcca
19	tgagagtaggatcgtatcca
20	gagtggaaaggaggggacag
21	tcatgtccctgttatataca
22	gcaggagtgcaagagataca
23	attaatggtccactagcagg
24	gcatatcctgatatagtgca
25	aaagaatgcggccttgttga
26	atcctttcaagtgcacagag
27	ctctttctgctttaaagcgg
28	attaatatgcctctggtgtc
29	caggtcatatgtgtaagggt
30	gtaggcatttcagaaccttt
31	ctcagcaacctctgcaataa
32	tgcctattaagagtcccaaa
33	ataaaccatccagaactgcc
34	aaactagcaggggatttgct
35	ctcttccttaatgatgggtt
36	aggcaaaagggatggcatga
37	agtcaagagaagggctttgc
38	aactgagacactgtagccat
39	catagggtgtttggggtaat
40	tgtaaaggaaagccccaagt
41	aaagtgctaagcttccttct
42	taacttttctggcagttggt
43	ttgcaatccaaatgcctttc
44	tcagtacactgagacactgc
45	agaaggcttaggtcatcttc
46	ttgccagagtttaatggtgg
47	ttctcagtaatgctgggaga
48	ctgcactggatgagttactt

Exon 7 Set	Oligo-probes
1	acaggcaccagagaaagtga
2	gagccatagctagtgaagac
3	agattatttctcagggcagt
4	gaccggacggaatgatgtat
5	acttcagagccacttgaatc
6	tcacaggtgtcctgtagaaa
7	tatgcttggacttagctcag
8	ctttatgggcaatggcaaca
9	agacccttgcacaatactat
10	caaatggctaagacccagtt
11	gatttagttccgtctcaagt
12	tatgcatgcttattcctagg
13	aggatggcagtatgcatatc
14	tttctctagatagcctgaca
15	tagaaaaccatacccctgctc
16	actactatgagcagggagtt
17	tcaggtacttagtctgatgc
18	tcagggcttgatcaagtaca
19	ttcatacgtttcctggttac
20	ggaagttacagtaggcttca
21	gcatgttattcctagagcaa
22	cctattttataggcagcttt
23	gtgcatgcctgggataaaag
24	acaaagctccacacagatct
25	gtggtgttaggtgataagca
26	tctatgtgttcgctcaacac
27	tttggtctagatttcactcc
28	taacatttagcacactgcct
29	ggaagtatcctagacagcat
30	tttactggcaaggtgtttgt
31	tcactctggccctataaaac
32	aggacctaggttagcatatt
33	aggaagtctccagtttatgt
34	ctgagccaggcaatgaacaa
35	ctgtagtctcaaggtgtgac
36	tgtttttcccagctaaagtg
37	ggaggggagcaactttttat
38	agtagcatcttctgcaatgt
39	ctgtaagtcacactgagtgc
40	ttcaacctctgaggcaaact
41	tccttgtagaacaggcgaac
42	agggcttctgtaactttagg
43	cttaatctgtttcattccca
44	agctctaaggcaacttggat
45	gtaacacttttgatgcccaa
46	gaaatgtaagcccattccta
47	gatgatggtaggatgtgctt
48	cagttggtgggaagatgact

Appendix Table S3 – Sequences of the two sets of Stellaris RNA FISH oligo-probes
