# Supplementary material for: The role of Xist‐mediated Polycomb recruitment in the initiation of X‐chromosome inactivation
Source: EMBO Rep. 2019 Aug 27;20(10):e48019. doi: 10.15252/embr.201948019 (PMC6776897; doi:10.15252/embr.201948019)
Supplement: Supplementary file 2 — Expanded View Figures PDF [file EMBR-20-e48019-s002.pdf]

Expanded View Figures

Figure EV1. Characterization of the novel *Xist*-TetOP mutants.

A Deletion mapping by Sanger sequencing and expression analysis across deleted regions in the novel *Xist* ΔF+B+C, ΔB+F, ΔB+C, ΔB+1/2C, ΔB, and ΔC mutants (this analysis is for clone 1 of each mutant type); the scheme represents only the first exon of *Xist*, and the color code for repeats matches the one on Fig 1A; red arrows indicate forward primers, and green arrows represent reverse primers; the primer on the left is the sequencing primer for each mutant; B means PCR blank.

B RT–PCR analysis of the splicing pattern and expression across the repeat B and C regions of the different *Xist*-TetOP mutants using the primer pairs indicated in the scheme in green (Appendix Table S2); B means PCR blank.

C *Xist* RNA FISH analysis upon D4 of differentiation in the presence of DOX (also noDOX for *Xist* FL) in the *Xist* ΔF+B+C, ΔB+F, ΔB+C, ΔB+1/2C, ΔB, and ΔC mutants (this analysis is for clone 1 of each mutant type); values represent the % ± SEM of cells with a *Xist*-coated chromosome (at least three biological replicates with a minimum of 250 cells counted per replicate; only two biological replicates for Xist FL noDOX); scale bar: 10 μm.

Source data are available online for this figure.

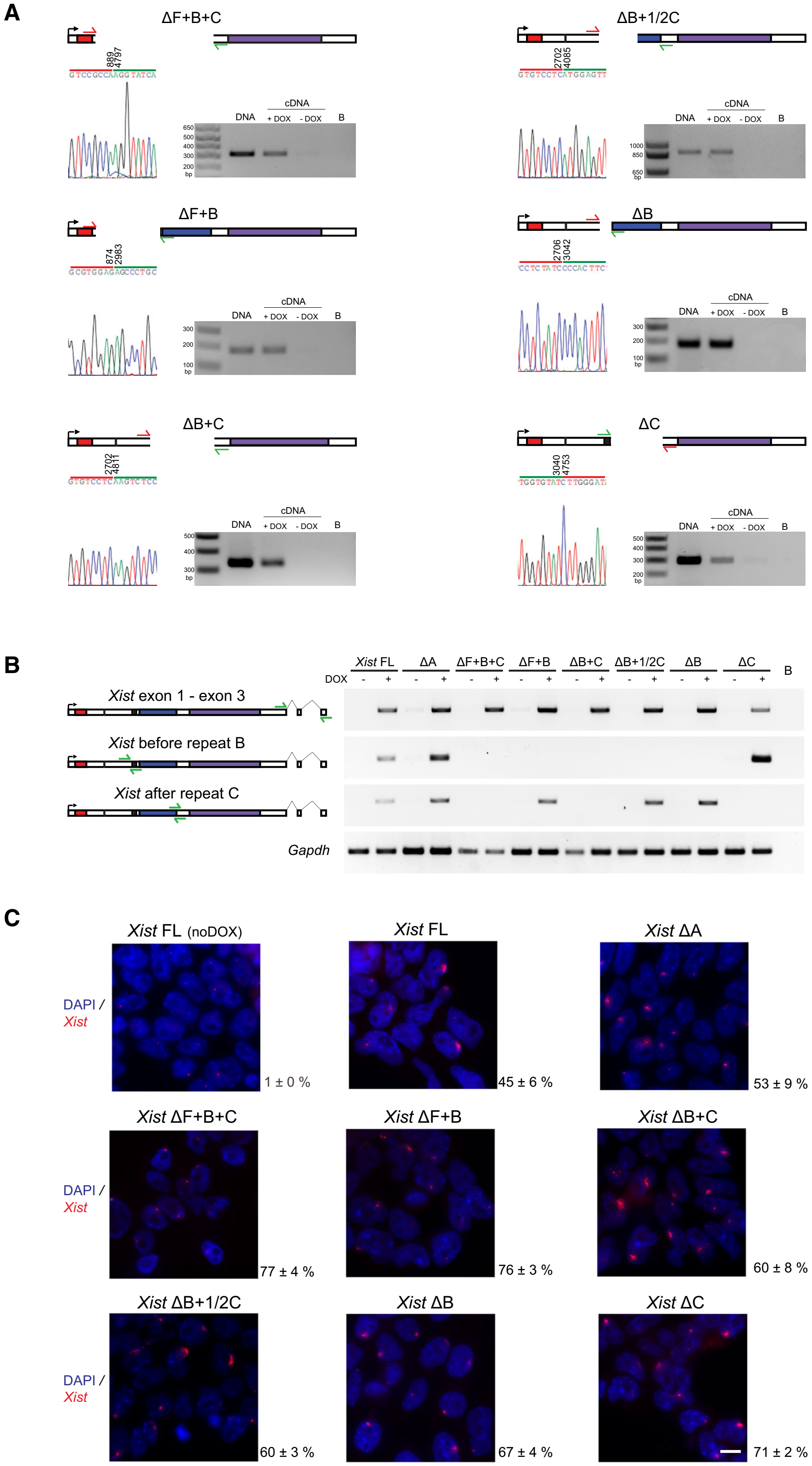

Figure EV1.

© 2019 The Authors

Figure EV2. Lack of PRC2 and PRC1 recruitment to the *Xist* ΔB+C-bound X chromosome is not caused by deficient chromosomal RNA coating.

A Representative images of combined IF for JARID2 (green) on the left and for RING1B (green) on the right with RNA FISH for *Xist* (red) in *Xist*-TetOP lines (for clone 1 of each mutant type) upon D2 in the presence of DOX; DAPI in blue; scale bar: 10 μm.

B Graph representing the mean % + SEM of *Xist*-coated chromosomes enriched for JARID2, EZH2, and RING1B in the different *Xist*-TetOP mutants (for clone 1 of each mutant type) from 2 to 4 independent experiments. A minimum of 50 *Xist*-coated chromosomes were counted per experiment. Only *P*-values corresponding to significant differences from unpaired Student's *t*-test, comparing mutants to *Xist* FL, are indicated as $*P < 0.05$.

C Normalized *Xist* RNA levels retrieved after actinomycin D treatment for 2, 4, and 6 h measured by RT–qPCR compared to 18S rRNA; shown are the mean of three independent biological replicates normalized to the non-treated conditions (0 h); errors bars represent SEM.

D Graph represents the mean area (above) and total intensity (below) of *Xist* RNA FISH signal in *Xist* FL and *Xist* ΔB+C-induced cells at day 2 of differentiation and in female MEFs; error bars represent SEM; a minimum of 71 *Xist* signals were counted per cell line; significant differences from unpaired Student's *t*-test, comparing mutants to *Xist* FL, are indicated as $*P < 0.05$ or $***P < 0.01$.

E Representative deconvoluted images from Z-projection images of RNA FISH using Stellaris fluorescent-labeled oligonucleotides targeting *Xist* in MEFs, *Xist* FL, and *Xist* ΔB+C-induced cells used for the quantification used in Fig EV3B; Scale bar: 10 μm.

Source data are available online for this figure.

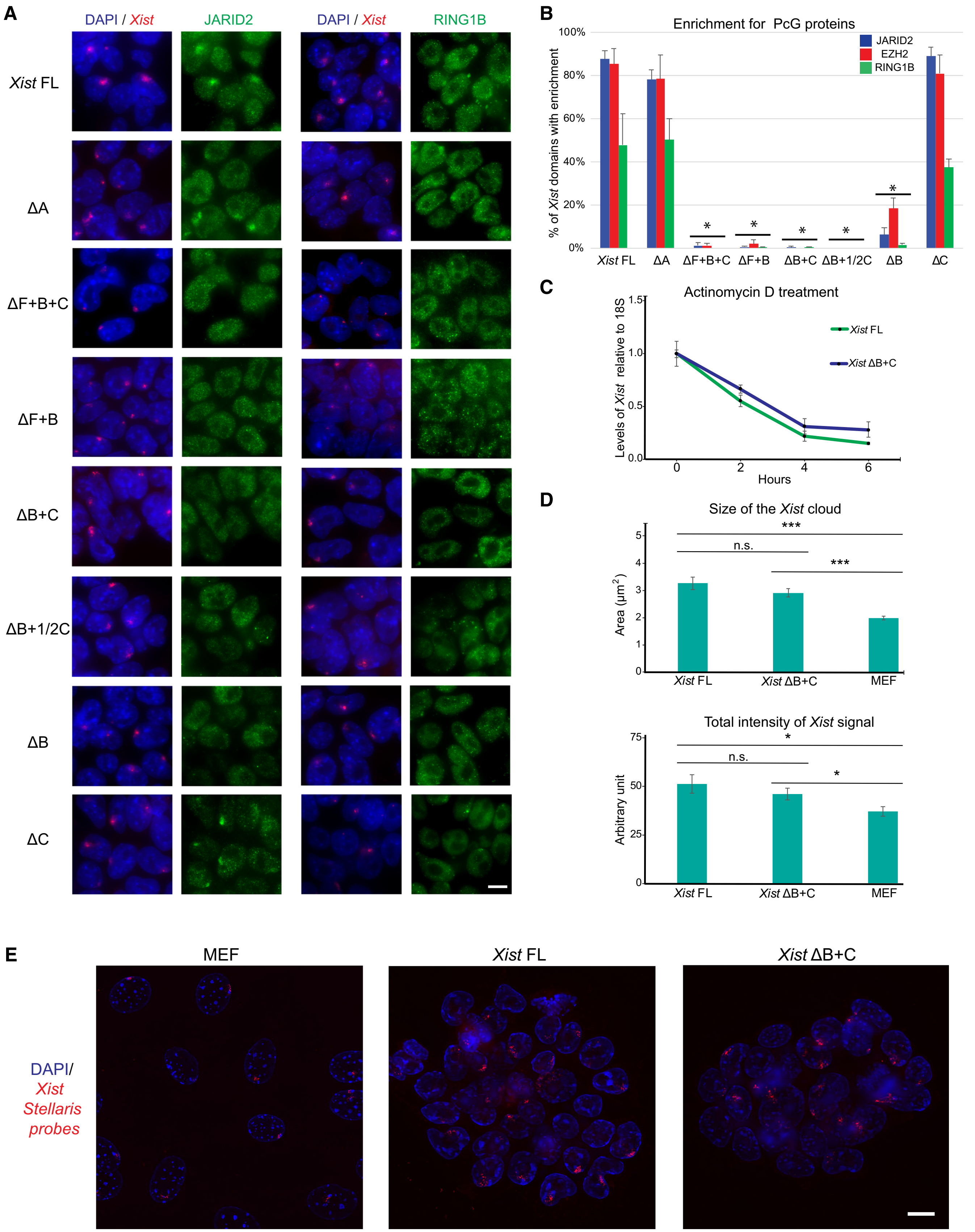

Figure EV2.

 © 2019 The Authors

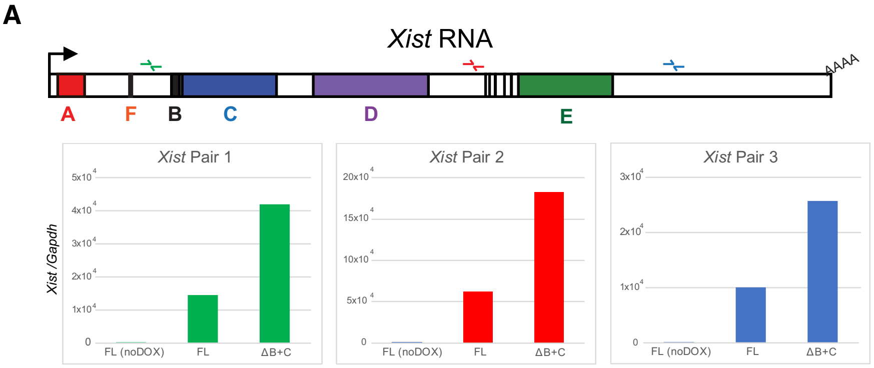

B

Samples	% of cells with *Xist* domains
Xist FL noDOX	0%
Xist FL DOX	24.0%
Xist ΔB+C DOX	50.9%

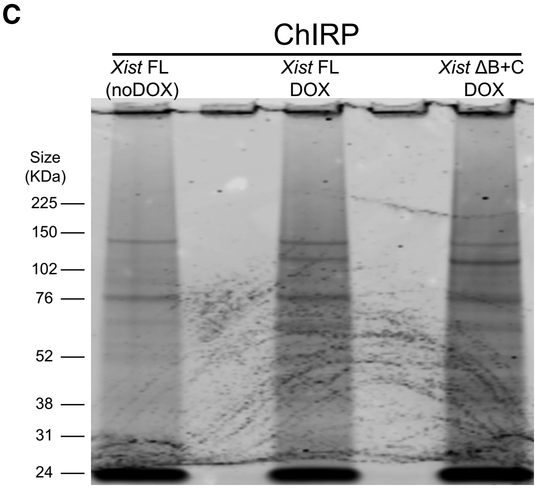

Figure EV3. Quality check of ChIRP procedure in *Xist* FL and *Xist* ΔB+C cells.

A RT–qPCR with three primer pairs along *Xist* to evaluate RNA retrieval after ChIRP procedure for *Xist* FL (in noDOX and DOX conditions) and *Xist* ΔB+C (DOX) at day 3 of differentiation.

B Table showing the percentage of cells exhibiting a *Xist*-coated X chromosome for *Xist* FL (both noDOX and DOX) and *Xist* ΔB+C (DOX) as determined by *Xist* RNA FISH used for ChIRP-MS; a minimum of 500 cells were counted.

C Blot visualized with Coomassie blue staining showing the band pattern of proteins displayed by *Xist* FL (both noDOX and DOX) and *Xist* ΔB+C (DOX) after ChIRP.

Figure EV4. Normalization of nChIP-seq to the percentage of *Xist*-induced cells in *Xist* FL and *Xist* ΔB+C confirms residual enrichment of PcG marks at initially active X-linked genes.

A Normalized signal of H3K27me3 and H2AK119ub around *HoxC* cluster (chr15: 102,840,000–103,110,000); shown is the signal of each sample around these cluster, normalized by the size of the library.

B Barplot representing percentages of H3K27me3 and H2AK119ub reads mapping on X chromosome (chrX) in each sample.

C Violin plots quantifying H3K27me3 and H2AK119ub enrichment over intergenic regions, initially active promoters, and initially active gene bodies on chrX and on autosomes in *Xist* FL and *Xist* ΔB+C cell lines upon DOX induction at day 2 of differentiation; shown is the distribution of the calculated log2 fold change in DOX versus noDOX conditions, the horizontal band is the median, and the lower and upper hinges correspond to the 25th and 75th percentiles; *P*-values were calculated using unilateral Wilcoxon test, comparing chrX and autosomal enrichment of PcG marks for each genomic region.

D Table showing the percentage of cells exhibiting a *Xist*-coated chrX for the different duplicates of *Xist* FL and *Xist* ΔB+C in DOX and noDOX conditions as determined by *Xist* RNA FISH; a minimum of 500 cells were counted to calculate the percentage of cells with a *Xist*-coated chrX.

E Violin plots quantifying H3K27me3 and H2AK119ub enrichment over intergenic regions, initially active promoters, and gene bodies on chrX in *Xist* FL and *Xist* ΔB+C upon DOX induction at day 2 of differentiation after normalization for the percentage of cells with *Xist*-coated chromosomes. Shown is the distribution of the calculated log2 fold change in DOX versus noDOX conditions, the horizontal band is the median, and the lower and upper hinges correspond to the 25th and 75th percentiles; *n* = indicates the number of genes analyzed; *P*-values were calculated using a paired Wilcoxon test, comparing *Xist* FL and *Xist* ΔB+C cell lines.

Source data are available online for this figure.

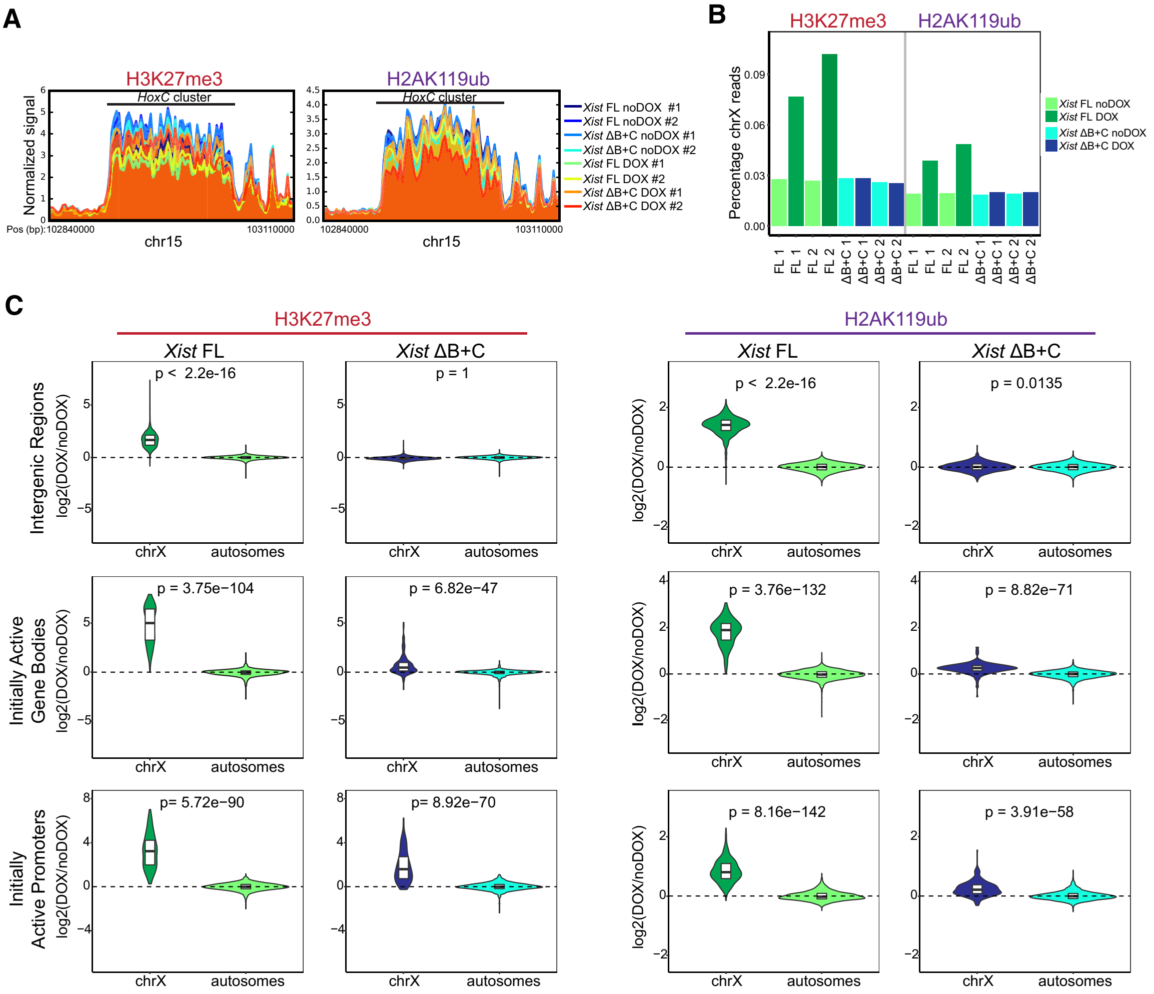

D

Samples	% of cells with *Xist* domains
Xist FL noDOX #1	0%
Xist FL noDOX #2	0%
Xist FL DOX #1	46.6%
Xist FL DOX #2	59.6%
Xist ΔB+C noDOX #1	0%
Xist ΔB+C noDOX #2	0%
Xist ΔB+C DOX #1	66.3%
Xist ΔB+C DOX #2	56.3%

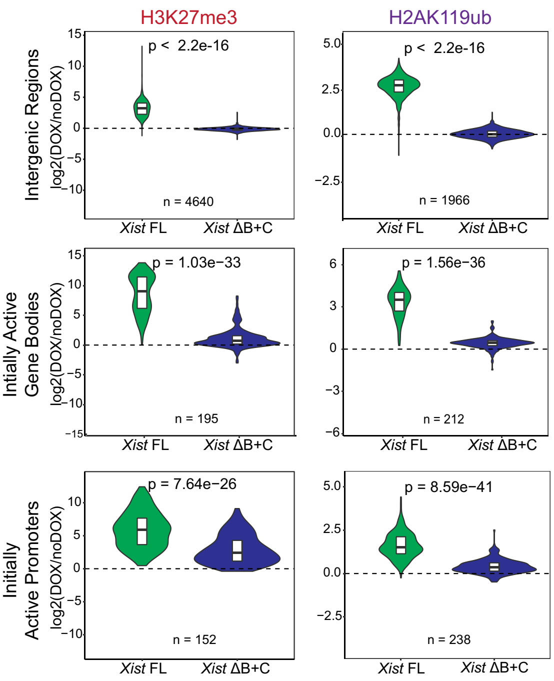

Figure EV4.

 © 2019 The Authors

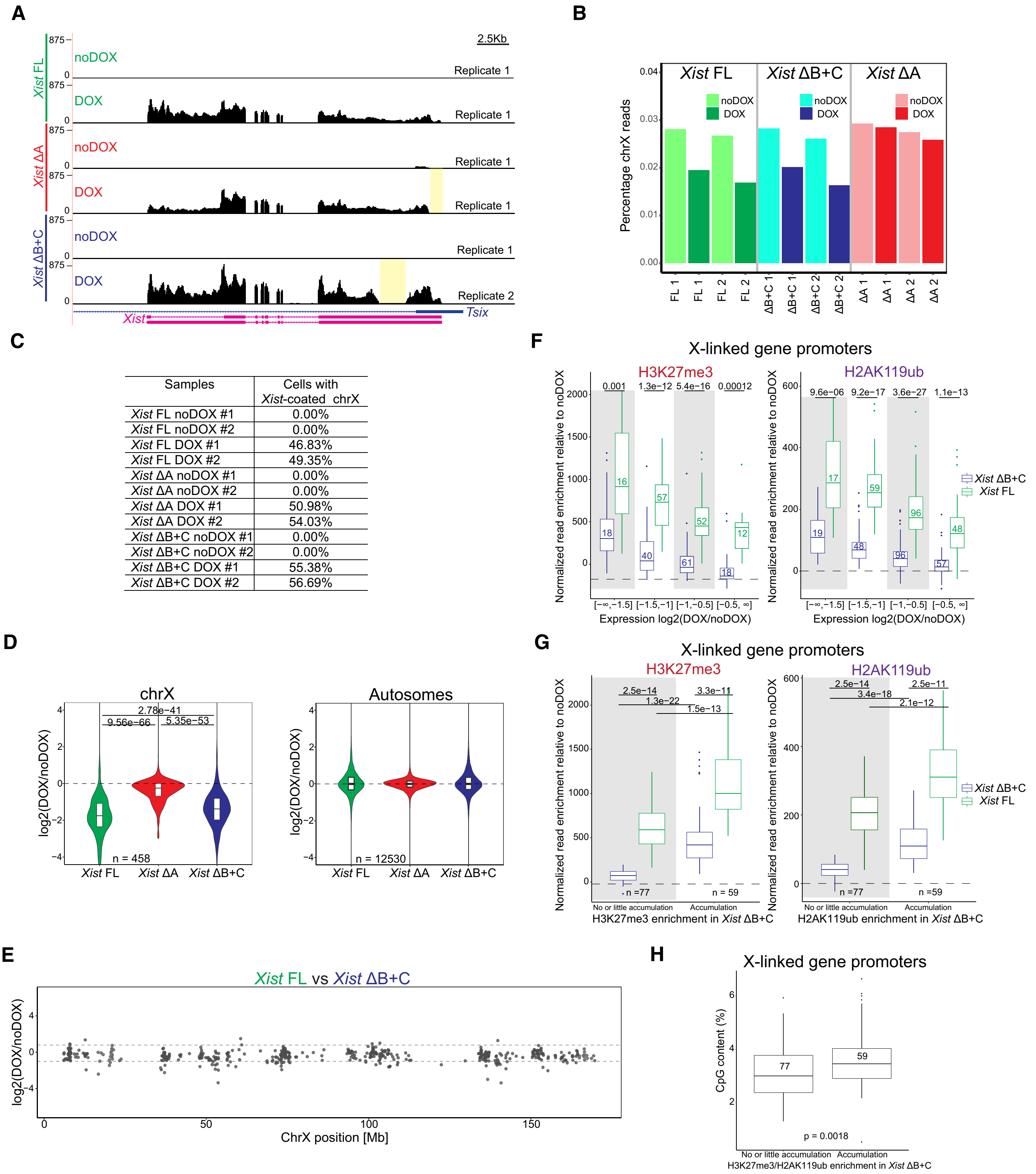

Samples	Cells with *Xist*-coated chrX
Xist FL noDOX #1	0.00%
Xist FL noDOX #2	0.00%
Xist FL DOX #1	46.83%
Xist FL DOX #2	49.35%
Xist ΔA noDOX #1	0.00%
Xist ΔA noDOX #2	0.00%
Xist ΔA DOX #1	50.98%
Xist ΔA DOX #2	54.03%
Xist ΔB+C noDOX #1	0.00%
Xist ΔB+C noDOX #2	0.00%
Xist ΔB+C DOX #1	55.38%
Xist ΔB+C DOX #2	56.69%

Figure EV5.

Figure EV5. Assessment of transcriptional changes by RNA-seq in *Xist* FL, *Xist* ΔA, and *Xist* ΔB+C-induced cells.

A Genome browser plots showing RNA-seq reads on *Xist/Tsix* genes for *Xist* FL, *Xist* ΔA, and *Xist* ΔB+C mutants in DOX and noDOX conditions at day 2 of differentiation; yellow boxes display the deleted regions in both *Xist* ΔA and *Xist* ΔB+C.

B Barplot representing percentages of RNA-seq reads mapping on X chromosome (chrX) in each sample.

C Table showing the percentage of cells exhibiting an *Xist*-coated chrX for the different duplicates of *Xist* FL, *Xist* ΔA, and *Xist* ΔB+C in DOX and noDOX conditions as determined by *Xist* RNA FISH; at least 500 cells were counted to estimate the percentage of cells with a *Xist*-coated chrX.

D Violin plots displaying the distribution of the average log2(fold change) in gene expression between DOX and noDOX conditions on chrX and autosomes in *Xist* FL, *Xist* ΔA, and *Xist* ΔB+C after normalization for the percentage of cells with a *Xist*-coated chrX; the horizontal band is the median of the values, and the lower and upper hinges correspond to the 25th and 75th percentiles; n = indicates the number of genes analyzed; *P*-values for chrX were calculated using a paired Wilcoxon test.

E Plots display the comparison of log2(fold change) in X-linked gene silencing upon DOX induction between *Xist* FL and *Xist* ΔB+C at day 2 of differentiation; Limma *t*-test did not find any gene differentially expressed between *Xist* FL and *Xist* ΔB+C.

F Box plots displaying the normalized read enrichment at promoters for H3K27me3 and H2AK119ub upon DOX induction for distinct categories of X-linked genes with different degrees of gene silencing between DOX and noDOX conditions in both *Xist* FL and *Xist* ΔB+C; the horizontal band of the box plot is the median of the values, the lower and upper hinges correspond to the 25th and 75th percentiles, the upper whisker extends from the hinge to the largest value not further than 1.5 interquartile range from the hinge, and the lower whisker extends from the hinge to the smallest value at most 1.5 interquartile range of the hinge; *P*-values were calculated using a Wilcoxon test; numbers inside the box plots indicate the number of genes analyzed.

G Box plots displaying H3K27me3 and H2AK119ub normalized enrichment levels at promoters upon induction in two categories of X-linked genes: with no or little accumulation versus with accumulation of these PcG marks in induced *Xist* ΔB+C cells; the horizontal band of the box plot is the median of the values, the lower and upper hinges correspond to the 25th and 75th percentiles, the upper whisker extends from the hinge to the largest value not further than 1.5 interquartile range from the hinge, and the lower whisker extends from the hinge to the smallest value at most 1.5 interquartile range of the hinge; *P*-values were calculated using a Wilcoxon test; n = indicates the number of genes analyzed.

H Box plots displaying the CpG content of promoters that accumulate or not H3K27me3/H2AK119ub between noDOX and DOX conditions in *Xist* ΔB+C at day 2 of differentiation; the horizontal band of the box plot is the median of the values, and the lower and upper hinges correspond to the 25th and 75th percentiles; the upper whisker extends from the hinge to the largest value not further than 1.5 interquartile range from the hinge, and the lower whisker extends from the hinge to the smallest value at most 1.5 interquartile range of the hinge; *P*-values were calculated using a Wilcoxon test; numbers inside the box plots indicate the number of promoters analyzed.

Source data are available online for this figure.

 © 2019 The Authors
