# Supplementary figures and images for: The role of Xist‐mediated Polycomb recruitment in the initiation of X‐chromosome inactivation
Source: EMBO Rep. 2019 Aug 27;20(10):e48019. doi: 10.15252/embr.201948019 (PMC6776897; doi:10.15252/embr.201948019)

source-data-Fig.EV1A

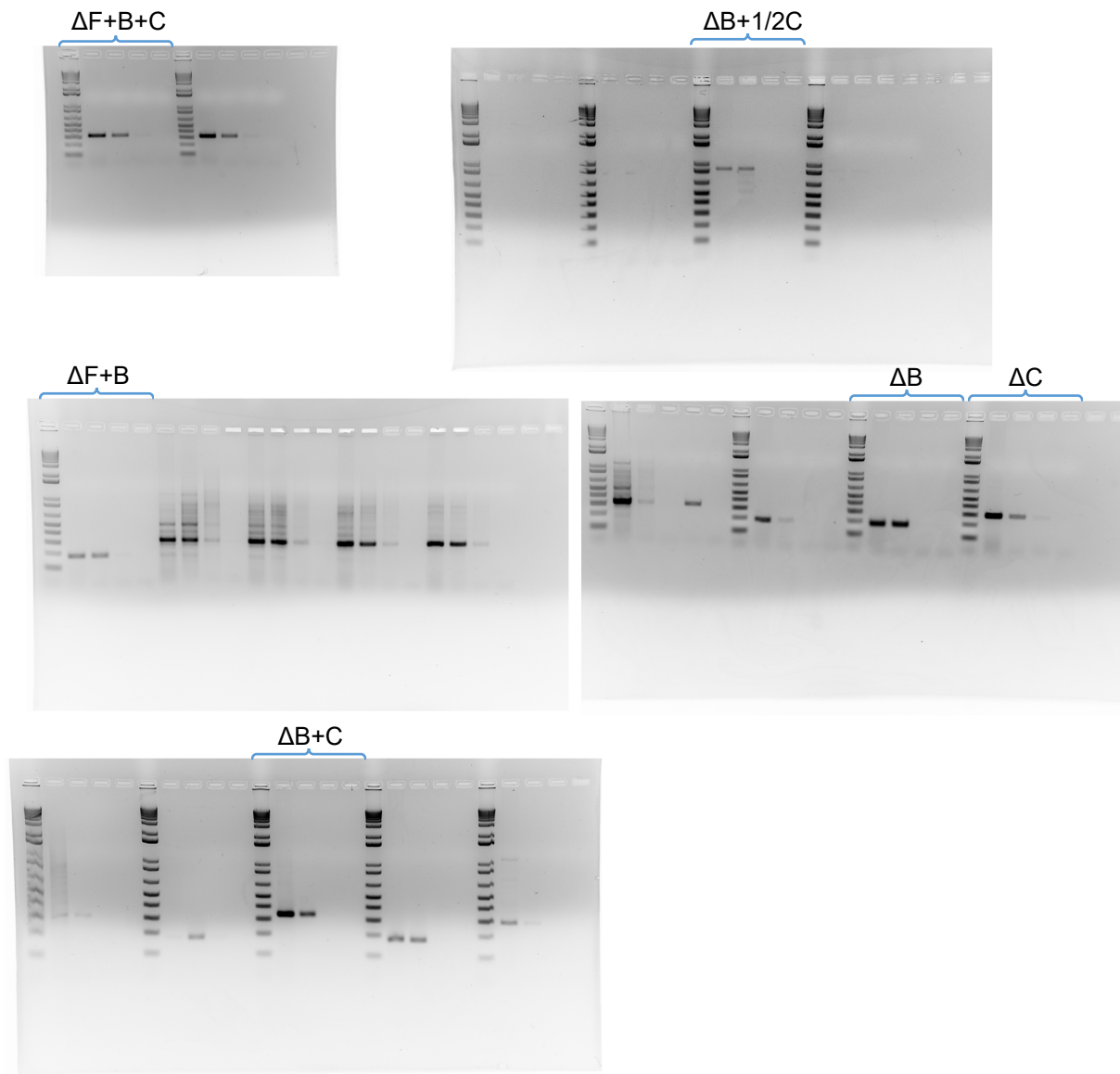

source-data-Fig.EV1B

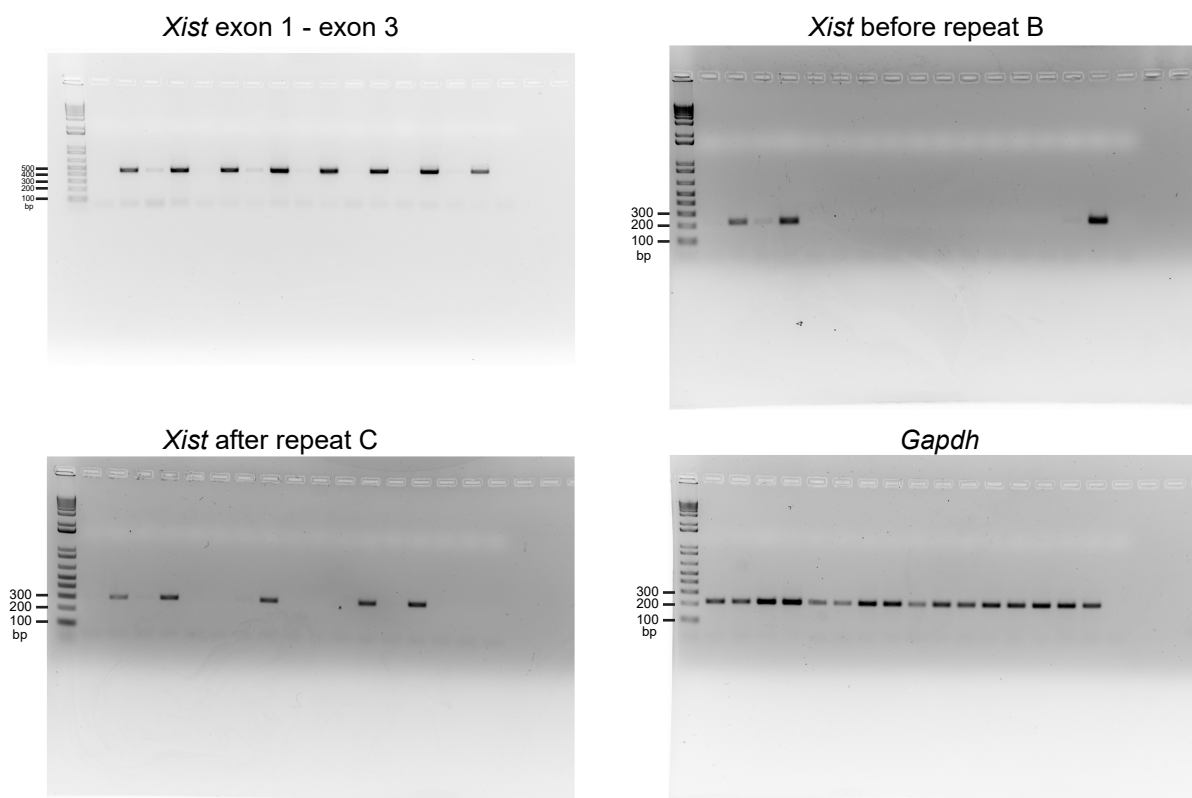

Supplement: Supplementary file 8 — Source Data for Expanded View [file EMBR-20-e48019-s011.zip › embr201948019-sup-0011-SDataEV/embr201948019-sup-0011-SDataFigEV1A-B.pdf]
